# Developmental Hip Dysplasia Treated with Cementless Total Hip Arthroplasty Using a Straight Stem and a Threaded Cup—A Concise Follow-Up, At a Mean of Twenty-Three Years

**DOI:** 10.3390/jcm10091912

**Published:** 2021-04-28

**Authors:** Viktor Janz, Christian Hipfl, Felix Düppers, Carsten F. Perka, Georgi I. Wassilew

**Affiliations:** 1 Department for Orthopaedic Surgery, University of Greifswald, 17475 Greifswald, Germany; georgi.wassilew@med.uni-greifswald.de; 2 Sporthopaedicum, 94315 Straubing, Germany; 3 Center for Musculoskeletal Surgery, Charité-Universitätsmedizin Berlin, 10115 Berlin, Germany; christian.hipfl@charite.de (C.H.); felix.dueppers@charite.de (F.D.); carsten.perka@charite.de (C.F.P.)

**Keywords:** DDH, THA, long-term follow-up, threaded cup

## Abstract

We previously reported the 9-year follow-up results of 121 cementless total hip arthroplasties (THAs) from 1990 to 1994 in 93 patients with developmental dysplasia of the hip (DDH). The present study reports the updated long-term results after a mean follow-up of 23 years. Fifty-seven patients (72 hips) were alive and available for follow-up. Since our previous report, nine THAs had been revised. The cumulative implant survivorship of any component was 87% (95% CI, 78–92%). The cumulative probability of not having aseptic cup loosening was 87% (95% CI, 77–93%) and there was no revision surgery for aseptic stem loosening. In three hips (5%), an exchange of the ball and liner due to polyethylene wear was performed after a mean of 12 years. This study demonstrates that cementless THA for DDH with restoration of the hip joint center provides excellent long-term durability.

## 1. Introduction

The osseous abnormalities of developmental dysplasia of the hip (DDH) represent a challenge to the correct reconstruction of the anatomical hip center. An anatomical reconstruction of the hip center is favorable to a high hip center, since this allows for an equalization of leg length and restoration of sufficient abductor strength [1,2,3].

One surgical treatment option to allow for a correct reconstruction of the anatomical hip center, in the presence of DDH, is the placement of a cementless small-diameter threaded cup in a medialized position within the true acetabulum. A medialized position of the acetabular cup has two advantages. First, the medialization allows for an optimal cranial coverage of the acetabular cup; secondly, a medialization of the hip center allows for a reduction in hip contact forces, which has been associated with a reduced rate of component loosening [4,5,6]. The disadvantage of this surgical treatment option is the thin PE liners, resulting from the small cup diameters, in young and highly active patients.

In our previous study, we reported the outcome of treatment of DDH with a cementless threaded cup and a cementless distally fixating Zweymüller-type stem in 121 patients with an average follow-up of nine years. In that study, survival rates of 97.5% and 100% for the acetabular and femoral components were reported, respectively [7].

The present study represents an update of the long-term results of this previously reported patient cohort treated with a cementless total hip arthroplasty (THA) for DDH after a mean follow-up of 23 years (range 22–25 years).

## 2. Materials and Methods

### 2.1. Study Subjects

We prospectively followed a previously published series of patients who were treated for DHH with a cementless THA at our institution from 1990 until 1994.

The original series included 121 hips in 93 DDH patients, 68% of whom were female. The mean age at the time of THA was 53 years (range 26–73 years). Since the previous report, 26 patients (33 hips) had died due to unrelated causes, seven hips (six patients) refused study participation, four hips (four patients) had a disclosure bar in our hospital IT system and five hips (three patients) were lost to follow-up, leaving 72 hips in 57 patients available for clinical and radiological follow-up. Overall, 13 hips (12 patients) underwent revision THA surgery. All deceased patients were free of revision surgery and had their THA in situ at the time of death.

### 2.2. Operative Technique

At the time of index surgery, a cementless Zweymüller System (Zimmer, Warschau, IN, USA), consisting of a threaded titanium cup paired with a grit-blasted, distally fixating, straight femoral stem with a ceramic head and an ultra-high molecular weight polyethylene liner, was utilized in all cases. All liners had a minimum thickness of 4.2 mm, were gamma radiated in an oxygen-free nitrogen environment and none of the liners had an elevated rim. All stems were without HA coating.

The aim of cup placement was to reconstruct the anatomical hip center. All threaded cups were reamed medially until a minimum of one thread was firmly anchored in the cranial acetabular bone stock. A medial perforation of the medial acetabular wall was accepted if this was necessary to engage at least one cranial thread of the cup. No structural bone grafts were performed in any acetabula. Additional femoral shortening osteotomies were performed in ten of the 17 hips with Crowe type IV DDH [8].

Initial postoperative radiographs were reviewed to determine the anatomical hip center, according to the method of Ranawat et al. [9]. Placement within 5 mm of this true anatomic hip center was regarded as correct implantation. Accordingly, the anatomical hip center of rotation was correctly reconstructed in 78 (66%) patients.

### 2.3. Radiological and Functional Assessment

In the present study, the radiological evaluation of all THAs was performed on conventional radiographs, pelvic overview and frog-leg lateral view of the affected hip by two authors (V.J. and F.D.) at the time of the clinical examination. Radiolucent lines or osteolysis were classified according to the DeLee and Charnley zones for the acetabular component and according to the Gruen zones for the femoral component [10,11,12]. Component loosening was defined as circumferential radiolucent lines in either projections or component migration over ≥3 mm. Acetabular component stability was assessed according to the method of Massin et al. and femoral component stability was assessed according to the method of Engh et al. [13,14].

The functional assessment of all THAs was performed via Harris hip score (HHS) and Merle d’Aubigné score by one of the authors (F.D.) at the time of follow-up [15,16]. Additionally, the patient’s subjective satisfaction with their THA was assessed as very good, good, satisfactory or unsatisfactory.

### 2.4. Statistical Analysis

Data are presented as mean values with ranges and compared using the Wilcoxon signed-rank test. Implant survivorship was calculated by the Kaplan–Meier method. Endpoints were defined as revision for any reason, revision of either the femoral or acetabular component and bearing exchange. A *p*-value of <0.05 was regarded as significant. Calculations were performed using GraphPad Prism (GraphPad software Inc., La Jolla, CA, USA).

## 3. Results

### 3.1. Implant Survivorship and Radiological Results 

In the previous study, at a mean mid-term follow-up of nine years, four failures were reported. Three cases of aseptic loosening (at four, eight and nine years postoperatively) and one case of delayed septic loosening (three months postoperatively) had occurred. This resulted in a nine-year survival rate for aseptic loosening of 97.5% and an overall survival rate of 96.7%. At the time of the first follow-up, no relationship between the degree of DDH, according to the classification of Crowe et al., and the rate of aseptic loosening was found [8].

Since our previous report, nine additional revisions had occurred for a total of 13 revision surgeries after a mean of 23 years (range 22–25 years). Overall, nine cases of aseptic cup loosening, three cases of polyethylene wear and one case of septic cup loosening were reported at the latest follow-up. The nine cases of aseptic cup loosening were treated with seven aseptic cup revisions and two aseptic THA revisions, due to simultaneous extensive femoral osteolysis. The three cases of polyethylene wear were treated with isolated ball and liner exchanges and the one case of septic cup loosening was treated with a one-stage septic THA exchange.

**Figure 1:**
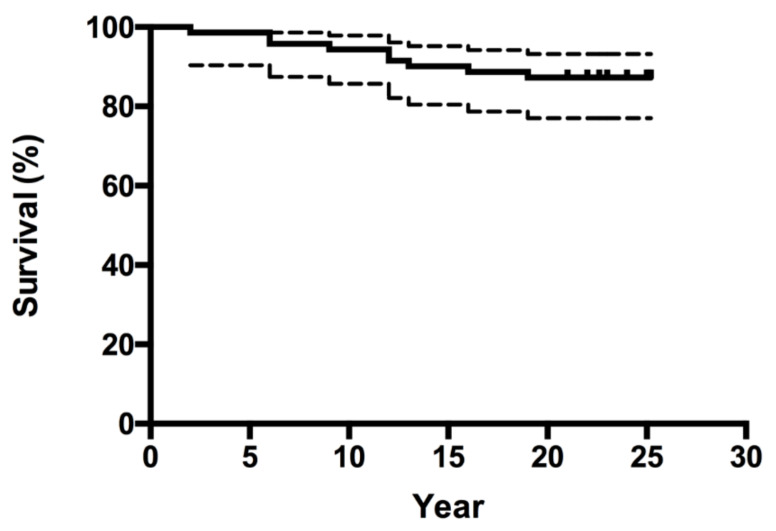
Kaplan–Meier survivorship curve (and corresponding 95% confidence intervals) for all seventy-two hips, with aseptic loosening of the cup as the end point.

**Figure 2:**
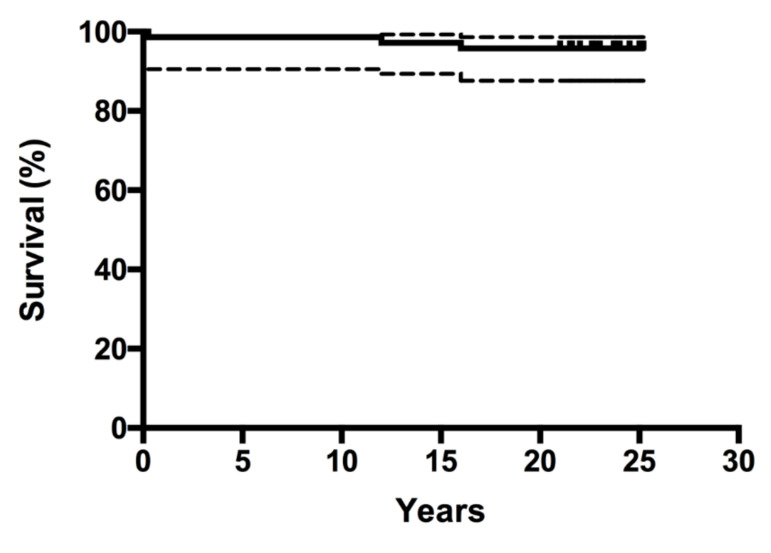
Kaplan–Meier survivorship curve (and corresponding 95% confidence intervals) for all seventy-two hips, with revision of the stem for any reason as the end point.

**Figure 3:**
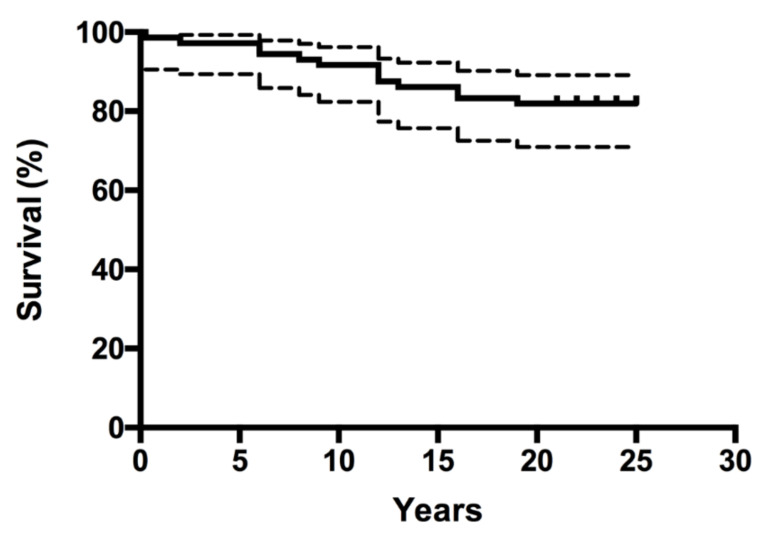
Kaplan–Meier survivorship curve (and corresponding 95% confidence intervals) for all seventy-two hips, with revision for any reason as the end point.

The Kaplan–Meier survivorship analysis for aseptic loosening of the acetabular component was 87.3% (95% CI, 77.1% to 93.2) at 23 years (Figure 1) and 86.1% (95% CI, 75.7% to 9239%) with revision for any reason as the endpoint. The Kaplan–Meier survivorship for aseptic loosening of the femoral component was 100% at 23 years, and 95.8% (95% CI, 87.6% to 98.6%) with revision for any reason as the endpoint (Figure 2). At 23 years, the overall implant survivorship with revision for any reason as the endpoint was 86.5% (95% CI, 77.8% to 91.9%) (Figure 3). In concordance with the results of our initial report, at nine years follow-up, still no relationship could be found between either the degree of DDH, according to the classification of Crowe et al., and the correct restoration of the anatomic hip center and the rate of aseptic component loosening at 23 years follow-up [8].

The radiological evaluation of the 59 unrevised hips revealed no continuous or progressive radiolucent lines or osteolysis around either the acetabular or femoral components. No THA was deemed at risk of loosening and thus, no indication for revision surgery was present at the 23-year follow-up.

### 3.2. Clinical Outcomes

In our initial report, the mean HHS improved from an average of 34.0 points (range 11–69) to 84.1 (range 29–100). After 23 years, the mean HHS of the 45 surviving patients (59 hips) remained significantly improved, with a mean score of 82.6 (range 41–100, *p* = 0.01) and the Merle d’Aubigné score also remained constant, with a mean of 15.3 points compared to 15.4 points at the nine-year follow-up. The subjective patient satisfaction also remained consistently high between the original and current follow-up; 80% of patients reported a very good, 9% a good and 11% a satisfactory outcome after 23 years.

Perioperative complications were reported in the previous follow-up; since then, no additional THA dislocations or periprosthetic fractures have occurred.

## 4. Conclusions

To our knowledge, this 23-year follow-up study represents the longest published follow-up data on cementless THA in DDH patients to date (Table 1). The cementless Zweymüller system demonstrated excellent durability in the clinical long-term follow-up. Even after 23 years, not a single stem was revised for aseptic loosening. Estimated THA survivorship, free from any component revision for any reason, was 86.5% (95% CI, 77.8% to 91.9%). The functional results remained excellent and unchanged between the previous report, at nine years follow-up, and the present study, at 23 years follow-up.

Our results are consistent with other studies reporting the outcome of cementless THA in DDH (Table 1. Nawabi et al. demonstrated a Kaplan–Meier survivorship for revision for any reason of 97% (95% CI, 79% to 99%) and no cases of aseptic loosening of the acetabular component in 27 hips at a mean follow-up of 12 years [17]. Murayama et al. reported 97% acetabular component survivorship after a mean of 15 years in 33 hips [18]. Kaneuji et al. found a 100% survivorship of the femoral component in 135 hips treated with a cementless THA at a mean follow-up of 14 years [19]. However, 17 cups (13%) had to be revised due to aseptic loosening or osteolysis in their series. In each of these reports, the authors accepted a slight superior placement of the acetabular component without using shelf grafts. In our study, in 78 hips (66%), the cup was placed in the anatomically correct center of rotation according to Ranawat et al. [9]. We did not find any differences regarding implant survival between patients with restoration of the anatomic hip center and those with a high placement of the acetabular component.

Our results are comparable to the long-term outcome reported for non-DDH patients treated with the cementless Zweymüller system [20,21]. Cruz-Pardos et al. found an overall component survival of 84% at 20 years, as well as a survival rate of 95% and 86% for the femoral and acetabular component, respectively [20]. Kolb et al. demonstrated an implant survivorship of 96% for the stem and 67% for the cup at a minimum of 20 years [21].

Our described surgical technique has some distinct biomechanical advantages over other surgical techniques, such as the use of shelf grafts to augment the deficient acetabular bone stock or an a priori acceptance of a high hip center [22]. Utilizing our described technique, encompassing the medial placement of a small-diameter cup in the true acetabulum, it is possible to medialize the hip center and thus, reduce the hip contact forces and resulting mechanical stress on the THA components [4]. Apart from the proven biomechanical advantages of a medialized hip center, Nawabie et al. were also able to show that a lateralized cup position was also associated with an increased PE wear in DDH patients [17].

**Table 1:** Overview of the recent literature in the treatment of DDH with cementless THA.

Author	No. of Hips	DDH Type	Mean Age (Years)	Implant Type	Mean FU (Years)	Survival Rate	Functional Results
Kaneuji et al. [23] (2009)	30	9 type I (30%) 17 type II (57%) 4 type III (13%)	53 (43–72)	HGP I	15.2 (10–18)	100% for the cup 100% for the stem	HHS 39 →85
Murayama et al. [18] (2012)	43	30 type I (70%) 11 type II (26%) 2 type III (4%)	55 (40–75)	Mallory-Head	15.3 (13–17)	97% for any reason	HHS 39 →85
Kaneuji et al. [19] (2013)	135	69 type I (51%) 57 type II (42%) 8 type III (6%) 1 type IV (1%)	49 (33–66)	HGP II HA/TCP Anatomic	13.5 (10–18)	89% for the cup 100% for the stem	HHS 51 →9
Nawabi et al. [17] (2014)	27	13 type II (48%) 14 type III (52%)	49 (28–77)	HA PSL S-ROM	12 (10–21)	97% for any reason 100% for the cup	HHS 36 →86 WOMAC 34 →84
Present Study (2018)	59	5 type I (9%) 19 type II (32%) 25 type III (42%) 7 type IV (12%)	53 (26–73)	Alloclassic	23 (22–25)	87% for any reason 91% for the cup 100% for the stem	HHS 34 →83 Merle d’Aubigné 9 →15

Polyethylene wear and acetabular osteolysis account for the most revisions in our series. This long-term problem has been reported by other authors for uncemented acetabular component and conventional ultra-high molecular weight polyethylene liners [17,19,20]. We believe that the use of newly highly cross-linked polyethylene will result in a decrease in liner wear and, consequently, in lower rates of aseptic loosening.

Our technique also offers a distinct advantage over the use of standard press-fit or cemented acetabular components in the presence of DDH. Both of these techniques are usually associated with the necessity of shelf grafting to compensate for the lack of cranial acetabular support. However, shelf grafts have a proven increased rate of failure if more than 25% of the cranial weight-bearing region is supported by the shelf graft [23,24].

The second advantage of our surgical technique is that a small-diameter cup can be used. This allows for an easy and quick surgical procedure, since the small diameter of the cup respects both the reduced a.p. diameter as well as the naturally hypoplastic anterior rim of the dysplastic acetabulum [25]. The use of a small-diameter threaded cup offers a quick reaming of the component bed, high primary component stability and circumvents the necessity for an additional shelf graft.

We demonstrated significant improvements in the HHS and Merle d’Aubigné score at the final follow-up. This is consistent with the functional results reported by other authors [17,18,19].

The authors acknowledge the limitations of this study. Firstly, although the data were prospectively collected, the patients were retrospectively reviewed. Secondly, as a result of the long-term follow-up and patient mortality, only 72 hips were available for complete clinical and radiological follow-up. This limitation is inherent to any clinical long-term follow-up study. Thus, our results must be interpreted in light of these facts.

In conclusion, THA in DDH using the cementless Zweymüller Alloclassic system shows excellent results in the long-term clinical follow-up. Radiographic changes in Gruen zone 1, around the shoulder of the femoral component, are relatively common, but without clinical relevance. The tapered design of the stem provides excellent stability and we recommend its continued use. However, polyethylene wear and osteolysis around the cup remain drawbacks and determine the long-term survival of this technique. The use of highly cross-linked polyethylene may contribute to even better future results related to wear and osteolysis in these young and active patients.

## Data Availability

Not applicable.
